# MRI and Clinical Variables for Prediction of Outcomes After Pediatric Severe Traumatic Brain Injury

**DOI:** 10.1001/jamanetworkopen.2024.25765

**Published:** 2024-08-05

**Authors:** Peter A. Ferrazzano, Susan Rebsamen, Aaron S. Field, Aimee T. Broman, Anoop Mayampurath, Bedda Rosario, Sandra Buttram, F. Anthony Willyerd, Paul J. Rathouz, Michael J. Bell, Andrew L. Alexander

**Affiliations:** 1 Department of Pediatrics, University of Wisconsin-Madison; 2 Waisman Center, University of Wisconsin-Madison; 3 Department of Radiology, University of Wisconsin-Madison; 4 Department of Biostatistics and Medical Informatics, University of Wisconsin-Madison; 5 Department of Epidemiology, School of Medicine, University of Pittsburgh, Pittsburgh, Pennsylvania; 6 Department of Child Health, Phoenix Children’s Hospital, Phoenix, Arizona; 7 Barrow Neurological Institute, Phoenix, Arizona; 8 Department of Population Health, Dell Medical School, The University of Texas at Austin, Austin; 9 Department of Pediatrics, Children’s National Medical Center, Washington, DC; 10 Department of Medical Physics, University of Wisconsin-Madison; 11 Department of Psychiatry, University of Wisconsin-Madison

## Abstract

**Importance:**

Traumatic brain injury (TBI) is a leading cause of death and disability in children, and predicting functional outcome after TBI is challenging. Magnetic resonance imaging (MRI) is frequently conducted after severe TBI; however, the predictive value of MRI remains uncertain.

**Objectives:**

To identify early MRI measures that predict long-term outcome after severe TBI in children and to assess the added predictive value of MRI measures over well-validated clinical predictors.

**Design, Setting, and Participants:**

This preplanned prognostic study used data from the Approaches and Decisions in Acute Pediatric TBI (ADAPT) prospective observational comparative effectiveness study. The ADAPT study enrolled 1000 consecutive children (aged <18 years) with severe TBI between February 1, 2014, and September 30, 2017. Participants had a Glasgow Coma Scale (GCS) score of 8 or less and received intracranial pressure monitoring. Magnetic resonance imaging scans performed as part of standard clinical care within 30 days of injury were collected at 24 participating sites in the US, UK, and Australia. Summary imaging measures were correlated with the Glasgow Outcome Scale–Extended for Pediatrics (GOSE-Peds), and the predictive value of MRI measures was compared with the International Mission for Prognosis and Analysis of Clinical Trials in TBI (IMPACT) core clinical predictors. Data collection, image analysis, and data analyses were completed in July 2023.

**Exposures:**

Pediatric severe TBI with an MRI scan performed as part of clinical care.

**Main Outcomes and Measures:**

All measures were selected a priori. Magnetic resonance imaging measures included contusion, ischemia, diffuse axonal injury, intracerebral hemorrhage, and brainstem injury. Clinical predictors included the IMPACT core measures (GCS motor score and pupil reactivity). All models adjusted for age and sex. Outcome measures included the GOSE-Peds score obtained at 3, 6, and 12 months after injury.

**Results:**

This study included 233 children with severe TBI who were enrolled at participating sites and had an MRI scan and preselected clinical predictors available. Their median age was 6.9 (IQR, 3.0-13.3) years, and more than half of participants (134 [57.5%]) were male. In a multivariable model including MRI measures and IMPACT core clinical variables, contusion volume (odds ratio [OR], 1.13; 95% CI, 1.02-1.26), brain ischemia (OR, 2.11; 95% CI, 1.58-2.81), brainstem lesions (OR, 5.40; 95% CI, 1.90-15.35), and pupil reactivity were each independently associated with GOSE-Peds score. Adding MRI measures to the IMPACT clinical predictors significantly improved model fit and discrimination between favorable and unfavorable outcomes compared with IMPACT predictors alone (area under the receiver operating characteristic curve, 0.77; 95% CI, 0.72-0.85 vs 0.67; 95% CI, 0.61-0.76 for GOSE-Peds score >3 at 6 months after injury).

**Conclusions and Relevance:**

In this prognostic study of children with severe TBI, the addition of MRI measures significantly improved outcome prediction over well-established and validated clinical predictors. Magnetic resonance imaging should be considered in children with severe TBI to inform prognosis and may also promote stratification of patients in future clinical trials.

## Key Points

**Question:**

What magnetic resonance imaging (MRI) findings are associated with long-term outcomes among children after severe traumatic brain injury (TBI), and does MRI improve outcome prediction over clinical measures alone?

**Findings:**

In this prognostic study of 233 children with severe TBI, MRI measures, including total contusion volume, total number of brain regions with ischemia, and presence of any brainstem lesion, were associated with outcome. The inclusion of MRI measures in predictive models significantly improved model performance over clinical predictors alone.

**Meaning:**

These findings suggest that MRI acquired within 30 days of pediatric severe TBI significantly improves outcome prediction.

## Teaser

This prognostic study identifies early magnetic resonance imaging (MRI) measures and investigates their association with prediction of long-term outcomes among children after severe traumatic brain injury (TBI).

## Introduction

Traumatic brain injury (TBI) is a leading cause of death and disability in children, affecting more than 3 million children worldwide each year^1^ and resulting in approximately 25 000 hospitalizations and 3000 deaths in the US alone.^2^ In the pediatric population, severe TBI frequently results in long-lasting neurocognitive and behavioral impairments with resultant effects on school performance, social functioning, and quality of life. Although some children recover completely from a severe TBI, long-term disability is common, with more than 60% of children requiring supportive services 1 year after injury^3^ and an estimated 145 000 children currently living with TBI-related disability.^4^ Given the high incidence of TBI and the wide range of functional outcomes after pediatric severe TBI, improved outcome prediction is critically important.

Predicting the degree of functional disability after severe TBI in children is challenging. Clinical factors such as Glasgow Coma Scale (GCS) scores and pupil reactivity have consistently been associated with outcome in both pediatric and adult TBI.^5,6,7,8^ These measures have been included in clinical scores such as the International Mission for Prognosis and Analysis of Clinical Trials in TBI (IMPACT) score to predict outcome after TBI.^9^ The IMPACT core measures of patient age, the motor component of the GCS (mGCS), and the Pupil Reactivity Score (hereinafter, pupil score) have recently been validated in a cohort of children with moderate and severe TBI.^10,11^ However, the added predictive value of additional clinical or neuroimaging measures in children with severe TBI remains unclear.

The enhanced resolution of magnetic resonance imaging (MRI) and increased sensitivity for detection of traumatic axonal injury, cytotoxic edema, and microhemorrhages^12^ make it a promising modality for potential outcome prediction after TBI. Magnetic resonance imaging is commonly performed during the acute hospitalization in children with severe TBI,^13^ and MRI measures have been associated with outcome after TBI in adults^14,15,16,17,18^ and children.^16,19,20,21,22,23^ These studies have been limited by small sample sizes, and they typically included a range of TBI severity and focused on single lesion types. Clinical studies have demonstrated improved outcome prediction with MRI measures over clinical variables alone in adults with mild TBI.^24^ Pediatric studies assessing the predictive value of acute MRI findings after severe TBI are limited; however, recent studies suggest that lesion volume, lesion depth, and diffuse axonal injury (DAI) assessed by MRI are associated with outcome^21,22,23^ in children with moderate and severe TBI. It remains unclear whether MRI measures acquired early after injury can improve prediction of long-term functional outcome after severe TBI in children.

The overall goals of this study were (1) to identify early MRI measures that predict long-term outcome after severe TBI in children and (2) to assess the added predictive value of MRI measures over the core IMPACT clinical variables. We collected and analyzed clinical MRI scans acquired within 30 days of injury in children with severe TBI and correlated summary imaging measures with the Glasgow Outcome Scale–Extended for Pediatrics (GOSE-Peds) assessment at 3, 6, and 12 months after injury.

## Methods

### Study Population

This prognostic study was performed in collaboration with the Approaches and Decisions in Acute Pediatric TBI (ADAPT) trial, which enrolled 1000 children (aged <18 years) with severe TBI (postresuscitation GCS score ≤8) in a prospective, observational study between February 1, 2014, and September 30, 2017.^25,26^ Twenty-four ADAPT clinical sites in the US, UK, and Australia participated in this MRI substudy^27^ and submitted the first brain MRI scan acquired within 30 days of injury for all ADAPT participants enrolled at their site who had an MRI scan performed as part of standard clinical care. No exclusions were made based on injury mechanism or other clinical factors. Therefore, this study includes consecutive patients with severe TBI at participating sites who had an MRI scan performed. All patients received intracranial pressure monitoring as a component of clinical care (intraparenchymal monitor or external ventricular drain, at the discretion of the treatment team). This study was approved by the institutional review boards at the University of Pittsburgh (ADAPT Coordinating Center), the University of Wisconsin (MRI Substudy Coordinating Center), and all participating sites. Written informed consent was obtained for outcome assessments from parents or guardians; when appropriate, assent was obtained from study participants. Participants who had an available pupil score at 12 hours after injury as well as an available postresuscitation mGCS score, who survived to hospital discharge, and who had at least 1 GOSE-Peds outcome assessment were included in our primary analysis of predictors of functional outcome. We focused this analysis on survivors due to the low number of participants who died and the potential for confounding by the expected use of MRI findings in clinical decision-making regarding discontinuation of life-sustaining support.^28^ This study followed the Transparent Reporting of a Multivariable Prediction Model for Individual Prognosis or Diagnosis (TRIPOD) reporting guideline.

### Clinical Measures, MRI Scanning, and Outcome Assessment

Patient demographic characteristics, injury characteristics, postresuscitation mGCS scores, and pupil scores (both reactive, 1 fixed, and both fixed) at 12 hours after admission were collected in the ADAPT study and used in this analysis. The MRI scanning protocols were determined by the clinical standard practice in use at each site. The MRI scans were read in a blinded fashion by 1 of 2 board-certified neuroradiologists (S.R. and A.S.F.), each with more than 20 years of clinical experience. Magnetic resonance imaging findings were coded according to the National Institute of Neurological Disorders and Stroke (NINDS) Common Data Elements (CDEs) for neuroimaging, using the lesion definition and brain region scheme as described by Haacke et al^29^ and detailed in the eMethods in Supplement 1. Before beginning the study readings, an interrater and intrarater reliability assessment was performed, and agreement between readers was found to be acceptable (eMethods and eTable 1 in Supplement 1). All scans were included in the analysis, and the best evaluation was made based on the experience and expertise of the neuroradiologist, given the clinical image quality, imaging protocol, and magnetic field strength. The GOSE-Peds is a global functional outcome measure with good concurrent, predictive, and discriminant validity^30^ and is included as an outcome assessment in the NINDS CDEs for pediatric TBI.^31^ The GOSE-Peds assessment consists of a structured, developmentally appropriate interview with the patient or caregiver and was conducted by telephone or in person by the ADAPT study team at each site at 3, 6, and 12 months after injury.

### Statistical Analysis

Demographic characteristics, clinical characteristics, imaging findings, and outcome scores were summarized using descriptive statistics. Our preselected set of MRI predictors included 5 quantified variables (total DAI microhemorrhage count, number of brain regions with DAI, total contusion volume, number of brain regions with contusion, and number of brain regions with ischemia) and 2 dichotomized variables (any brainstem lesion and any intracerebral hemorrhage [ICH]). Our a priori clinical predictors included the mGCS and the pupil score (IMPACT core predictors). A log_2_ transformation was applied to the quantified variables to enhance interpretation (outcome effect is associated with a 2-fold increase in the predictor variable), with a measure of 0 represented as log_2_ of one-half of the lowest positive number for that variable.

Univariate associations of the selected variables with the longitudinal GOSE-Peds score were determined using an ordinal logistic regression random-effects modeling framework, in which each patient was allowed to have their own random intercept, adjusting for age, sex, and GOSE-Peds time point. The outcome assessment time point was included in the repeated-measures model to allow for the overall trend of GOSE-Peds score over time. Our framework adjusted for longitudinal changes in GOSE-Peds score, facilitated each patient having their individual starting intercept, and incorporated patients with at least 1 GOSE-Peds measurement.

We then used a multivariable ordinal logistic regression random-effects modeling framework to determine the association between our primary outcome and clinical and MRI measures. Similar to the univariate models, we allowed each patient to have their own random effect for intercept and adjusted for age, sex, and GOSE-Peds time point. We derived and compared 3 models: a clinical-only model (mGCS and pupil score), an MRI-only model (number of brain regions affected by axonal injury, total volume of contusion, number of brain regions affected by ischemia, presence or absence of any ICH, and presence or absence of any brainstem injury), and a clinical plus MRI model (including all of the aforementioned clinical and MRI measures). Diffuse axonal injury total lesion count and number of regions with contusion were not included in multivariable models owing to the high correlation with number of regions with DAI and total contusion volume, respectively. The fit of the 3 multivariable models was compared using the Akaike information criterion (AIC), in which a model with the lowest AIC indicates the model that best explains the variance in outcome with the fewest predictors. Hochberg^32^ adjustment of *P* values to preserve family-wise error rate was used across univariate models. *P* < .05 (2-tailed) was considered statistically significant.

Finally, we determined the ability of each model to predict neurological outcomes at 3, 6, and 12 months. Briefly, we dichotomized our numerical GOSE-Peds score at each time point into favorable (GOSE-Peds score ≤3; upper good recovery to upper moderate disability) vs unfavorable (GOSE-Peds score of 4-7; lower moderate disability to vegetative) binary outcomes. We derived clinical-only, MRI-only, and clinical plus MRI logistic regression models using the aforementioned features and evaluated each model’s ability to discriminate between favorable and unfavorable outcomes at 3, 6, and 12 months. Our primary metric for this analysis was the area under the receiver operating characteristic curve (AUROC). Repeated cross-validation, in which we considered 20 permutations with different seeds of 10-fold cross-validation, was used to create 200 AUROC out-of-sample estimates. We used 1000 bootstrap samples to determine the 95% CIs for the AUROC values.

All analyses were performed with R, version 4.1.0 (R Project for Statistical Computing).^33^ Ordinal logistic regression was performed using the ordinal package, version 2019.12.15.^34^ Repeated-measures regression was performed using the lme4 package, version 1.1-26.^35^ Area under the curve (AUC) was calculated using the pROC package, version 1.18.^36^ Natural splines for the age predictor were calculated using the splines package, version 2.0-7.^33^ Finally, we created folds for the cross-validation using the caret package, version 6.0-88.^37^ Data collection, image analysis, and data analyses were completed in July 2023.

## Results

### Demographics and Injury Characteristics

A total of 233 patients were included in our primary analysis of associations between MRI measures and functional outcome. Their median age was 6.9 (IQR, 3.0-13.3) years; 134 patients (57.5%) were male and 99 (42.5%) were female (Table 1). Initially, there were 355 patients at 24 sites with a clinical MRI scan performed during the first 30 days after injury. Of these patients, 253 had both an mGCS score and a pupil score available and they also had at least 1 GOSE-Peds score. Characteristics of excluded patients are shown in eTable 2 in Supplement 1. Twenty patients died during hospitalization and were analyzed separately, and no patients died after discharge from the hospital. In a planned secondary analysis, univariate associations between imaging findings and death were determined and are presented in eTable 3 in Supplement 1. A participant selection flow diagram is shown in the eFigure in Supplement 1. The most common lesion was contusion (186 [79.8%]), followed by DAI (168 [72.1%]), ischemia (82 [35.2%]), and ICH (38 [16.3%]). Brainstem lesions were also common, occurring in 91 patients (39.1%) (eTable 6 in Supplement 1).

**Table 1.** Patient Characteristics

Characteristic	Participant (N = 233)^a^
Age, y	6.9 (3.0-13.3)
Females	99 (42.5)
Males	134 (57.5)
TBI cause	
Any motor vehicle–related injury	139 (59.7)
Fall	40 (17.2)
Inflicted	32 (13.7)
Unknown or other	22 (9.4)
TBI type	
Closed	213 (91.4)
Penetrating or crush	20 (8.6)
Abuse	
No concern	190 (81.5)
Possible	11 (4.7)
Probable	16 (6.9)
Definite	16 (6.9)
TBI mechanism	
Acceleration or deceleration	21 (9.0)
Impact	162 (69.5)
Crush	6 (2.6)
Fall	36 (15.5)
Gunshot	5 (2.1)
Unknown or other	3 (1.3)
Total GCS score	6 (3-7)
mGCS score	3 (1-4)
Pupil score	
Both reactive	180 (77.3)
1 Fixed	28 (12.0)
Both fixed	25 (10.7)
MRI time point after injury, h	134.55 (62.58-211.00)
Diffuse axonal injury	168 (72.1)
Lesion count	34.5 (16.75-61.25)
Region count	6 (3-9)
Contusion	186 (79.8)
Total volume, cm^3^	18.14 (4.27-54.84)
Region count	3 (2-4)
Ischemia	82 (35.2)
Region count	4 (2-6.8)
Intracerebral hemorrhage	38 (16.3)
Brainstem injury	91 (39.1)
Outcome assessment time point	
3 mo (n = 210)	
GOSE-Peds score	4 (3-6)
Unfavorable outcome	112 (53.3)
6 mo (n = 224)	
GOSE-Peds score	3 (2-6)
Unfavorable outcome	96 (42.9)
12 mo (n = 162)	
GOSE-Peds score	3 (2-6)
Unfavorable outcome	73 (45.1)

Abbreviations: GCS, Glasgow Coma Scale; GOSE-Peds, Glasgow Outcome Scale–Extended for Pediatrics; mGCS, motor component of the Glasgow Coma Scale; MRI, magnetic resonance imaging; TBI, traumatic brain injury.

^a^ Unless indicated otherwise, values are presented as the median (IQR) or the number (percentage) of participants.

### Association of MRI Measures With Outcome

Univariable ordinal logistic regression models were used to determine the association of GOSE-Peds score with clinical and MRI measures, adjusting for age, sex, and GOSE-Peds assessment time point (Table 2). Total contusion volume was associated with the GOSE-Peds score (odds ratio [OR], 1.19; 95% CI, 1.05-1.33), with a 2-fold increase in contusion volume associated with approximately 20% increase in odds of having a higher GOSE-Peds score (worse outcome). Ischemia was associated with GOSE-Peds score (OR, 2.28, 95% CI, 1.68-3.11), with a 2-fold increase in the number of regions affected by ischemia associated with a 2.3-fold increase in the likelihood of a higher GOSE-Peds score. The presence of brainstem injury (OR, 8.44; 95% CI, 3.19-22.30) was also associated with an approximately 8-fold increased odds of having a higher GOSE-Peds score. Both DAI and ICH demonstrated a borderline association with GOSE-Peds score. The pupil score was associated with the GOSE-Peds score (both fixed: OR, 47.29; 95% CI, 10.55-211.90), and an inverse association was found between mGCS and GOSE-Peds scores. Small associations were found between GOSE-Peds scores and DAI, ICH, and mGCS score.

**Table 2.** Univariate Associations of Clinical and MRI Measures With GOSE-Peds Scores^a^

Measure	OR (95% CI)	*P* value
**MRI variables**		
DAI		
Total lesion count	1.22 (1.03-1.45)	.052
Total region count	1.33 (1.00-1.76)	.052
Contusion		
Total volume	1.19 (1.05-1.33)	.02
Total region count	1.46 (1.00-2.13)	.052
Ischemia		
Total region count	2.28 (1.68-3.11)	<.001
ICH present	3.72 (1.06-13.10)	.052
Brainstem lesion present	8.44 (3.19-22.30)	<.001
**Clinical variables**		
mGCS score	0.71 (0.53-0.94)	.02
Pupil score		
1 Fixed	1.79 (0.47-6.80)	<.001
Both fixed	47.29 (10.55-211.90)	

Abbreviations: DAI, diffuse axonal injury; GOSE-Peds, Glasgow Outcome Scale–Extended for Pediatrics; ICH, intracerebral hemorrhage; mGCS, motor component of the Glasgow Coma Scale; MRI, magnetic resonance imaging; OR, odds ratio.

^a^ Mixed ordinal logistic regression was performed, adjusted for age, sex, and GOSE-Peds time point.

In a mixed multivariable ordinal logistic regression including only the clinical variables (clinical-only model) and adjusting for age, sex, and GOSE-Peds time point, both mGCS score and pupil score were independently associated with GOSE-Peds score. In the model including only the MRI measures (MRI-only model), contusion volume, number of regions with ischemia, and brainstem injury were independently associated with increased GOSE-Peds score. After accounting for all other MRI measures, DAI and ICH were not associated with GOSE-Peds score. In the model including both MRI measures and clinical variables (clinical plus MRI model), contusion (OR, 1.13; 95% CI, 1.02-1.26), ischemia (OR, 2.11; 95% CI, 1.58-2.81), brainstem injury (OR, 5.40; 95% CI, 1.90-15.35), and pupil score (1 fixed vs both fixed: 1.74; 95% CI, 0.51-6.00 vs 10.12; 95% CI, 2.45-41.84; *P* = .005) were associated with GOSE-Peds score, whereas mGCS score was no longer associated with GOSE-Peds score after accounting for all other clinical and MRI measures (Table 3).

**Table 3.** Multiple-Predictor Models for GOSE-Peds Score^a^

Model	OR (95% CI)	*P* value	AIC
**Clinical only**			
mGCS score	0.75 (0.57-0.99)	.04	1795
Pupil score			
1 Fixed	1.74 (0.46-6.57)	<.001	
Both fixed	41.69 (9.40-184.94)		
**MRI only**			
DAI, total region count	1.00 (0.75-1.24)	.98	1770
Contusion, total volume	1.17 (1.05-1.30)	.005	
Ischemia, total region count	2.28 (1.70-3.04)	<.001	
ICH present	1.47 (0.46-4.64)	.51	
Brainstem lesion present	7.90 (2.76-22.60)	<.001	
**Clinical plus MRI**			
DAI, total region count	1.00 (0.75-1.33)	.99	1763^b^
Contusion, total volume	1.13 (1.02-1.26)	.02	
Ischemia, total region count	2.11 (1.58-2.81)	<.001	
ICH present	1.06 (0.34-3.33)	.92	
Brainstem lesion present	5.40 (1.90-15.35)	.001	
mGCS score	0.85 (0.66-1.09)	.19	
Pupil score			
1 Fixed	1.74 (0.51-6.00)	.005	
Both fixed	10.12 (2.45-41.84)		

Abbreviations: AIC, Akaike information criterion; DAI, diffuse axonal injury; GOSE-Peds, Glasgow Outcome Scale–Extended for Pediatrics; ICH, intracerebral hemorrhage; mGCS, motor component of the Glasgow Coma Scale; MRI, magnetic resonance imaging; OR, odds ratio.

^a^ Mixed ordinal logistic regression, adjusted for age, sex, and GOSE-Peds time point.

^b^ Significant improvement over clinical-only model (*P* < .001).

### Model Performance

As presented in Table 3, the addition of MRI measures significantly improved model performance over the clinical-only model (AIC, 1763 vs 1795; *P* < .001). Additionally, the MRI-only model performed favorably compared with the clinical-only model. The power of each model to predict a favorable outcome (GOSE-Peds score ≤3) vs an unfavorable outcome (GOSE-Peds score of 4-7) at 3, 6, and 12 months after injury was assessed for the clinical-only, MRI-only, and clinical plus MRI models using cross-validated ROC curves. As shown in the Figure, the AUROC values for the MRI-only model were greater than those for the clinical-only model at each time point, and the predictive power of each model was greatest for the 6-month GOSE-Peds time point. The AUC values were not significantly different between the MRI-only model and the clinical plus MRI model. The AUC for the MRI-only model and for the clinical plus MRI model was significantly greater than that for the clinical-only model for both the 6-month (0.76; 95% CI, 0.71-0.84 and 0.77; 95% CI, 0.72-0.85, respectively, vs 0.67; 95% CI, 0.61-0.76) and 12-month (0.71; 95% CI, 0.64-0.80 and 0.69; 95% CI, 0.62-0.80, respectively, vs 0.58; 95% CI, 0.48-0.72) GOSE-Peds time points (eTables 4 and 5 in Supplement 1), with the largest improvement in discrimination for the 12-month time point (AUC difference, 0.134; 95% CI, 0.005-0.234).

**Figure.**
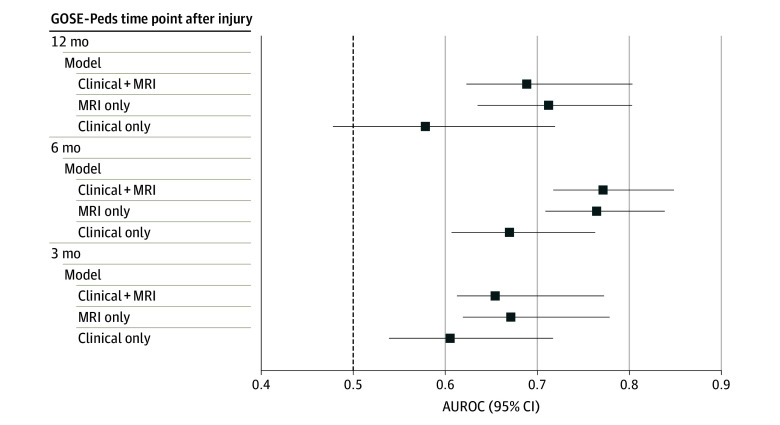
Predictive Model Area Under the Receiver Operating Characteristic Curve (AUROC) Comparisons The discriminative ability of each model for favorable or unfavorable outcome (Glasgow Outcome Scale–Extended for Pediatrics [GOSE-Peds] score of 1-3 vs 4-7) is shown for 3, 6, and 12 months after injury. The mean AUROC was determined in a 10-fold cross-validation with 20 random partitions of the data, and the 95% CI was determined in 1000 bootstrap samples. The dashed vertical line at 0.5 AUROC indicates a discriminative ability no better than random assignment. MRI indicates magnetic resonance imaging.

## Discussion

In this study of children with severe TBI who survived to hospital discharge and had an MRI scan performed within 30 days of injury, we found that MRI measures of ischemia, contusion, and brainstem injury were independently associated with the GOSE-Peds score after adjusting for age, sex, mGCS score, and pupil reactivity. We also determined that the addition of MRI measures significantly improved model fit and the prediction of 6-month and 12-month unfavorable outcome.

### MRI Findings Associated With Outcome

Not surprisingly, contusion was the most common lesion type encountered in our severe TBI population, and contusion volume was associated with a modest but statistically significant increase in odds of a worse outcome. This finding is consistent with prior studies that have demonstrated an association between lesion volume and functional outcome in any-severity TBI in children.^20,38,39,40^ The predictive ability of contusion volume in children with severe TBI is much less well established. In a study of 67 children with moderate and severe TBI, Smitherman et al^21^ found that fluid-attenuated inversion recovery (FLAIR) lesion volume was associated with GOSE-Peds score. In our large cohort of children with severe TBI, we found that a doubling of total contusion volume on MRI was associated with an approximately 20% increase in the likelihood of a higher GOSE-Peds score. We also found a high incidence of ischemic lesions on MRI in our study, and the number of brain regions with ischemia was associated with outcome. Ischemia may occur as a direct result of the inciting traumatic injury during the immediate peri-injury period or secondary to vascular injury^41^ or inadequate cerebral perfusion during the acute hospitalization. Episodes of cerebral hypoxia/ischemia are common after pediatric severe TBI, occurring in up to 50% of children,^42^ and avoiding this secondary injury underlies many clinical management strategies after severe TBI.^43^ Ischemic injury has been associated with worse outcome in adults^44^ and children with severe TBI.^45^ In our study, we found that a 2-fold increase in the number of brain regions with ischemia was associated with an approximately 2-fold increase in the likelihood of being in a higher GOSE-Peds category.

The presence of any brainstem lesion was associated with an increase in the GOSE-Peds score. The majority of brainstem lesions in our study participants were microhemorrhages associated with DAI, with additional contribution from ischemia, contusion, and hemorrhage. In contrast, the overall brain burden of DAI (total number of DAI lesions and total number of regions with DAI) was not associated with outcome, highlighting the importance of lesion depth. Depth of injury has long been recognized as a marker of TBI severity and forms the basis for the DAI grading system (0 indicates no DAI; 1, subcortical DAI; 2, corpus callosum DAI; and 3, brainstem DAI).^46,47^ When applied to cohorts with any-severity TBI, the DAI grade has been associated with outcome^48^; however, this association is less clear in pediatric severe TBI.^23^ In a large single-center study of 136 children with severe TBI, Janas et al^22^ found that although hemorrhagic DAI grade was not associated with outcome, the depth of diffusion restriction lesions was associated with time to command following. Smitherman et al^21^ extended the DAI grade paradigm to assess the association between FLAIR lesion depth and outcome, and they found that brainstem lesions were associated with a worse GOSE-Peds score compared with other lesion patterns. The association between brainstem injury and outcome after TBI is well established.^49^ In a 2017 meta-analysis of 27 studies examining the prognostic value of MRI, brainstem injury was found to increase the risk of mortality (risk ratio, 1.78) and unfavorable GOS (risk ratio, 2.49).^50^ In a recent study of 43 children with severe TBI who underwent craniectomy and subsequent MRI scanning, Baker et al^51^ demonstrated that the presence of FLAIR lesions and microhemorrhages in the brainstem was associated with poor outcome. Importantly, these FLAIR lesions represent a number of pathologies, including contusion, axonal injury, ischemia, or edema.^52,53,54^ In our study, the presence of any brainstem lesion in the participants was independently associated with an approximately 8-fold increase in the likelihood of having a higher GOSE-Peds score.

### Added Predictive Power of Neuroimaging Over the IMPACT Core Clinical Measures

The large public health burden of TBI, the heterogeneity of brain injury mechanisms and severity, and the wide range of potential outcomes have motivated many efforts to develop clinically useful prediction models. Two predictive models have been developed from large adult datasets of patients with moderate and severe TBI: the Corticosteroid Randomization After Significant Head Injury (CRASH) model^55^ and the IMPACT model.^9,56^ These models have undergone extensive external validation in adult cohorts with moderate to severe TBI^57,58,59,60^ and, more recently, in pediatric cohorts with TBI.^10,11^ The IMPACT model includes a core set of predictors that contains most of the prognostic information^9^: age, mGCS, and pupil reactivity. These clinical variables are similar to those in the CRASH model, and they are consistent with the large body of work that has established the association between these clinical measures and outcome after TBI in adults and children.^5,6,7,8^

Magnetic resonance imaging has been viewed as a promising tool for improving outcome prediction after TBI, but few studies have assessed the additional predictive power provided by MRI. In a study from the TRACK-TBI Investigators, MRI was found to improve prediction of unfavorable outcome at 3 months after mild TBI.^24^ In a large pediatric severe TBI cohort, Janas et al^22^ found that adding the number and depth of brain regions with magnetic resonance diffusion restriction lesions improved model prediction of time to command following and the discharge score on the Functional Independence Measure for Children. Our overall goal was to determine whether MRI measures improved outcome prediction; therefore, we compared MRI measures with the IMPACT core variables of mGCS and pupil reactivity and we adjusted for age across all models. We found that the MRI-only model performed favorably compared with the clinical-only model, and the addition of MRI measures to the clinical predictors significantly improved model performance over the clinical-only model. When we compared the ability of each model to discriminate between favorable and unfavorable outcomes by using cross-validated AUROC values for GOSE-Peds score at 3, 6, and 12 months after injury, we found that the MRI-only and MRI plus clinical models performed significantly better than the clinical-only model, and MRI models were most predictive of outcome at 6 months after injury.

### Strengths and Limitations

Strengths of our study include the multisite study design, large sample size, well-defined severe TBI population, inclusion of all injury mechanisms, and use of the NINDS CDEs for neuroimaging. Additionally, we used the NINDS CDEs for neuroimaging to create the largest dataset to date, to our knowledge, of clinical MRI findings in children with severe TBI.

There are also some limitations that should be recognized when interpreting the results from our study. All MRI scans were performed as part of clinical care, leading to heterogeneity in timing of MRI and imaging protocols, which may have affected the associations between imaging measures and outcome. Decisions on whether to obtain an MRI scan were at the discretion of the clinicians, so care should be taken in generalizing our findings to the overall population of children with severe TBI. Future prospective studies would benefit from the use of standardized imaging criteria, protocols, and time points. Two neuroradiologists assessed the entire cohort of MRI scans using the well-established NINDS CDE definitions for neuroimaging. This standardized approach may not reflect MRI interpretations typically performed as part of clinical care. Our primary outcome (GOSE-Peds score) was a measure of overall function, so our results should not be extended to specific neuropsychological or cognitive deficits, which may each variably affect functioning. In our cohort of children with severe TBI, the clinical-only model underperformed compared with what has been described previously, which may be accounted for by the inclusion of patients with nonsevere TBI in those studies. Finally, the limited sample size prevents us from reliably making conclusions regarding outcome uncertainty.

## Conclusions

To our knowledge, this prognostic study is the first to establish the added value of clinical MRI findings in predicting long-term outcome after severe TBI in children. Contusion volume, number of ischemic brain regions, and presence of any brainstem lesion were each independently associated with outcome. Furthermore, MRI measures significantly improved model performance and prediction of an unfavorable outcome at 6 and 12 months after injury. These findings suggest that MRI scanning should be considered to inform prognosis after severe TBI in children. Inclusion of MRI scanning in future pediatric TBI clinical trials may allow for improved stratification of patients. Additional research is needed to define the optimal timing of MRI scanning after TBI, and to determine the evolution of MRI findings over the course of postinjury brain development.

## Notes

Supplement 1.
eMethods.
**eTable 1.** Interrater and Intrarater Reliability **eTable 2.** Patient Characteristics (Included vs Excluded) **eTable 3.** Univariate Association of Clinical and Magnetic Resonance Imaging Measures With Death **eTable 4.** Predictive Model AUROCs for Unfavorable Outcome **eTable 5.** AUROC Differences Between Each Model **eTable 6.** Summary of Brainstem Lesion Types (n = 91) **eFigure.** Patient Flow Diagram

Supplement 2.
Nonauthor Collaborators

Supplement 3.
Data Sharing Statement
