# Supplementary material for: MRI and Clinical Variables for Prediction of Outcomes After Pediatric Severe Traumatic Brain Injury
Source: JAMA Netw Open. 2024 Aug 5;7(8):e2425765. doi: 10.1001/jamanetworkopen.2024.25765 (PMC11301548; doi:10.1001/jamanetworkopen.2024.25765)
Supplement: Supplement 1. — eMethods. eTable 1. Interrater and Intrarater Reliability eTable 2. Patient Characteristics (Included vs Excluded) eTable 3. Univariate Association of Clinical and Magnetic Resonance Imaging Measures With Death eTable 4. Predictive Model AUROCs for Unfavorable Outcome eTable 5. AUROC Differences Between Each Model eTable 6. Summary of Brainstem Lesion Types (n = 91) eFigure. Patient Flow Diagram [file jamanetwopen-e2425765-s001.pdf]

Supplementary Online Content

Ferrazzano PA, Rebsamen S, Field AS, et al; ADAPT MRI Investigators. MRI and clinical variables for prediction of outcomes after pediatric severe traumatic brain injury. *JAMA Netw Open*. 2024;7(8):e2425765. doi:10.1001/jamanetworkopen.2024.

eMethods.

eTable 1. Interrater and Intrarater Reliability

eTable 2. Patient Characteristics (Included vs Excluded)

eTable 3. Univariate Association of Clinical and Magnetic Resonance Imaging Measures With Death

eTable 4. Predictive Model AUROCs for Unfavorable Outcome

eTable 5. AUROC Differences Between Each Model

eTable 6. Summary of Brainstem Lesion Types (n = 91)

eFigure. Patient Flow Diagram

This supplementary material has been provided by the authors to give readers additional information about their work.

eMethods

MRI scanning and analysis

The first clinical brain MRI scan obtained within 30 days of injury was assessed. The MR scanning protocols were determined by the clinical standard practice in use at each site, and therefore varied across subjects. MRI scans were read in a blinded fashion by one of two board certified neuroradiologists, each with >20 years of clinical experience, and coded according to the NIH/NINDS Common Data Elements (CDEs) for Neuroimaging. Brain regions assessed included bilateral frontal, parietal, temporal, and occipital lobes, bilateral thalamus/basal ganglia and internal capsule, and the cerebellum and brainstem. DAI was additionally assessed in the corpus callosum. For measurement of contusion volume and intracerebral hemorrhage (ICH) volume, the greatest extent of each lesion was measured in 3 orthogonal planes and an idealized ellipsoid volume was calculated (ABC*pi/6). Summary contusion measures included the total contusion volume summed across all brain regions, and the number of brain regions with any contusion. ICH was defined as a collection of confluent, relatively homogeneous blood within the brain parenchyma, and quantified as the total lesion volume in cubic centimeters (hemorrhage and surrounding edema). For quantification of DAI, microhemorrhages in each region were counted up to a total of 10 lesions, and regions with >10 lesions received a score of 11. The summary DAI measures included the total microhemorrhage count summed across all brain regions, and the total number of brain regions with any microhemorrhage. Ischemia was defined as evidence of tissue injury on diffusion-weighted imaging (DWI) consistent with a deficit between substrate demand and delivery. Ischemia was scored as present/absent in each brain region and quantified as the number of brain regions affected by that lesion type, and lesion volume was not assessed. Brainstem injury was scored as presence or absence of any lesion within the brainstem, including microhemorrhage, contusion, ischemia, or edema (eTable 6). Analysis was restricted to intraparenchymal lesions due to the high prevalence of invasive procedures in the population (external ventricular drain, craniectomy) which make assessment of extra-axial fluid/blood unreliable. Furthermore, MRI changes deemed to be clearly related to invasive procedures/devices (small hemorrhages/edema along EVD tracks, etc.) were not included in the analyses. All scans were included in the analysis, and the best evaluation was made based on the experience and expertise of the neuroradiologist given the clinical image quality, imaging protocol, and magnetic field strength. Upon completion of the inter-rater reliability assessment, the neuroradiologists were able to confer with each other for the subsequent study reads. If a lesion or location could not be assessed, it was considered not present/uninjured in subsequent analyses.

Assessment of inter- and intra-rater reliability

Forty MRI scans were randomly selected by stratifying the 355 subjects into 20 groups by age, sex and participating site, and selecting 2 scans at random from each group. Each neuroradiologist read the 40 scans in a blinded fashion and coded the findings according to the NIH/NINDS Common Data Elements for Neuroimaging. After a 6 week washout period, one scan from each of the 20 groups was randomly selected and each neuroradiologist reread those scans. Cohens Kappa was calculated (weighted and unweighted) to assess inter- and intra-rater reliability. Quantitative measures were binned according to tertiles (33rd and 66th percentiles) of the positive measurements summed over all locations, with zero serving as a separate category, yielding scores of 0, 1, 2, or 3. Kappa weights

© 2024 Ferrazzano PA et al. *JAMA Network Open.*

were specified as 1.0 for an exact match, 0.0 for discordance between a 0 and any value (>0), or between any two values differing by >1. To formally assess statistical uncertainty, we calculated ordinary bootstrapped confidence intervals (n=10000 bootstrap re-samples of the 40 scans) for both the unweighted and weighted kappa statistics.Intra-rater reliability was calculated as the mean of the two within-grader kappa (unweighted and weighted) statistics (eTable 1).

eTable 1. Interrater and Intrarater Reliability

**Inter-rater Reliability**				
	Presence/Absence		Quantification	
	Kappa	95% CI	Weighted kappa	95% CI
Contusion	0.58	0.23, 0.84	0.50	0.30, 0.69
DAI	0.88	0.55, 1.00	0.71	0.51, 0.85
ICH	0.75	0.42, 0.94	0.65	0.41, 0.85
Ischemia	0.61	0.36, 0.84	0.54	0.34,0.72
Brainstem Lesion	0.94	0.65, 1.00	0.76	0.57, 0.91
**Intra-rater Reliability**				
	Presence/Absence		Quantification	
	Kappa	95% CI	Weighted kappa	95% CI
Contusion	0.81	0.00, 1.00	0.79	0.54, 0.93
DAI	0.80	0.00, 1.00	0.70	0.53, 0.85
ICH	0.68	0.41, 0.88	0.69	0.43, 0.89
Ischemia	0.61	0.36, 0.84	0.54	0.34,0.72
Brainstem DAI	0.75	0.44, 0.95	0.64	0.34, 0.81

DAI, diffuse axonal injury; ICH, intracerebral hemorrhage; Quantifications: contusion, volume (cc); DAI, total number of lesions; ICH, volume (cc); ischemia, number of regions affected.

© 2024 Ferrazzano PA et al. *JAMA Network Open.*

eTable 2. Patient Characteristics (Included vs Excluded)

		Included (N=233)	Excluded (N=102)
Age (years), median [IQR]		6.88 [2.99, 13.33]	4.26 [1.37,12.27]
Sex, n (%)	Female	99 (42.5%)	37 (36.3%)
	Male	134 (57.5%)	65 (63.7%)
TBI Cause, n (%)	MVA	139 (59.7%)	50 (49%)
	Fall	40 (17.2%)	16 (15.7%)
	Inflicted	32 (13.7%)	28 (27.5%)
	Unknown/Other	22 (9.4%)	8 (7.8%)
TBI Type, n (%)	Closed	213 (91.4%)	95 (93.1%)
	Penetrating/Crush	20 (8.6%)	7 (6.9%)
Abuse, n (%)	No concern	190 (81.5%)	69 (67.6%)
	Possible	11 (4.7%)	10 (9.8%)
	Probable	16 (6.9%)	12 (11.8%)
	Definite	16 (6.9%)	11 (10.8%)
TBI Mechanism, n (%)	Acceleration/Deceleration	21 (9%)	12 (11.8%)
	Impact	162 (69.5%)	66 (64.7%)
	Crush	6 (2.6%)	2 (2%)
	Fall	36 (15.5%)	15 (14.7%)
	Gunshot	5 (2.1%)	4 (3.9%)
	Unknown/Other	3 (1.3%)	3 (3%)
Total GCS score, median [IQR]		6 [3, 7]	3.5 [3,6]
GCS motor score, median [IQR]		3 [1, 4]	3 [1,4]
Pupil Score, n (%)	Both reactive	180 (77.3%)	72 (70.6%)
	One fixed	28 (12%)	13 (12.7%)
	Both fixed	25 (10.7%)	13 (12.7%)
MRI time (hours post-injury), median [IQR]		134.55 [62.58, 211]	164.57 [59.44,243.07]
Diffuse Axonal Injury, n (%)		168 (72.1%)	68 (66.7%)
Lesion count, median [IQR]		34.5 [16.75, 61.25]	22 [8,56]
Region count, median [IQR]		6 [3, 9]	4 [2,8]
Contusion, n (%)		186 (79.8%)	73 (71.6%)
Total volume (cc), median [IQR]		18.14 [4.27,54.84]	30.17 [9.44,71.16]
Region count, median [IQR]		3 [2,4]	3 [2,4]
Ischemia, n (%)		82 (35.2%)	43 (42.2%)
Region count, median [IQR]		4 [2, 6.75]	5 [2,9]
Intracerebral Hemorrhage, n (%)		38 (16.3%)	15 (14.7%)
Brainstem Injury, n (%)		91 (39.1%)	39 (38.2%)

© 2024 Ferrazzano PA et al. *JAMA Network Open.*

eTable 3. Univariate Association of Clinical and Magnetic Resonance Imaging Measures With Death

	OR	95% CI
Diffuse Axonal Injury, total lesion count	0.92	(0.78, 1.09)
Diffuse Axonal Injury, total region count	0.87	(0.66, 1.15)
Contusion, total volume	0.99	(0.88, 1.11)
Contusion, total region count	0.89	(0.62, 1.30)
Ischemia, total region count	1.64	(1.21, 2.26)
Any intracerebral hemorrhage	1.18	(0.31, 3.70)
Any Brainstem Injury	1.99	(0.73, 5.56)
GCS Motor Score	0.57	(0.38, 0.80)
Pupil Score, one fixed	0.71	(0.04, 4.05)
Pupil Score, both fixed	8.60	(3.12, 24.4)

Logistic Regression, Adjusted for Age and Sex

eTable 4. Predictive Model AUROCs for Unfavorable Outcome

	3 Months	6 Months	12 Months
Clinical-only	0.605 [0.540, 0.716]	0.670 [0.608, 0.762]	0.578 [0.48, 0.718]
MRI-only	0.671 [0.620, 0.778]	0.764 [0.710, 0.838]	0.712 [0.636, 0.802]
Clinical+MRI	0.654 [0.614, 0.772]	0.771 [0.719, 0.848]	0.689 [0.624, 0.803]

Cross-validated AUROC for 1000 bootstrap samples was used to determine the 95% confidence interval of the cross-validated AUROC for GOSE-Peds at 3, 6, and 12 months post-injury. AUROC, area under the receiver operator characteristic curve; [95% CI]; unfavorable outcome, GOSE-Peds 4-7.

eTable 5. AUROC Differences Between Each Model

	3 Months	6 Months	12 Months
MRI-only – Clinical-only	0.066 [-0.011, 0.159]	0.094 [0.011, 0.165]	0.134 [0.005, 0.234]
Clinical+MRI – Clinical-only	0.049 [-0.003, 0.149]	0.101 [0.0135, 0.165]	0.111 [0.011, 0.220]
Clinical+MRI – MRI-only	-0.017 [-0.027, 0.028]	0.007 [-0.013, 0.039]	-0.023 [-0.033, 0.037]

Differences between bootstrapped AUROC models were taken for each bootstrapped permutation, and an overall estimate of the AUROC difference and 95% bootstrapped confidence interval was estimated at each post-injury timepoint. AUROC, area under the receiver operator characteristic curve; [95% CI].

eTable 6. Summary of Brainstem Lesion Types (n = 91)

Injury	N (%)
Diffuse Axonal Injury	86 (94.5)
Contusion	2 (2.2)
Ischemia	2 (2.2)
Intracerebral hemorrhage	1 (1.1)
Injury/edema NOS	3 (3.3)

NOS, not otherwise scored with other lesion types.

© 2024 Ferrazzano PA et al. *JAMA Network Open.*

eFigure. Patient Flow Diagram

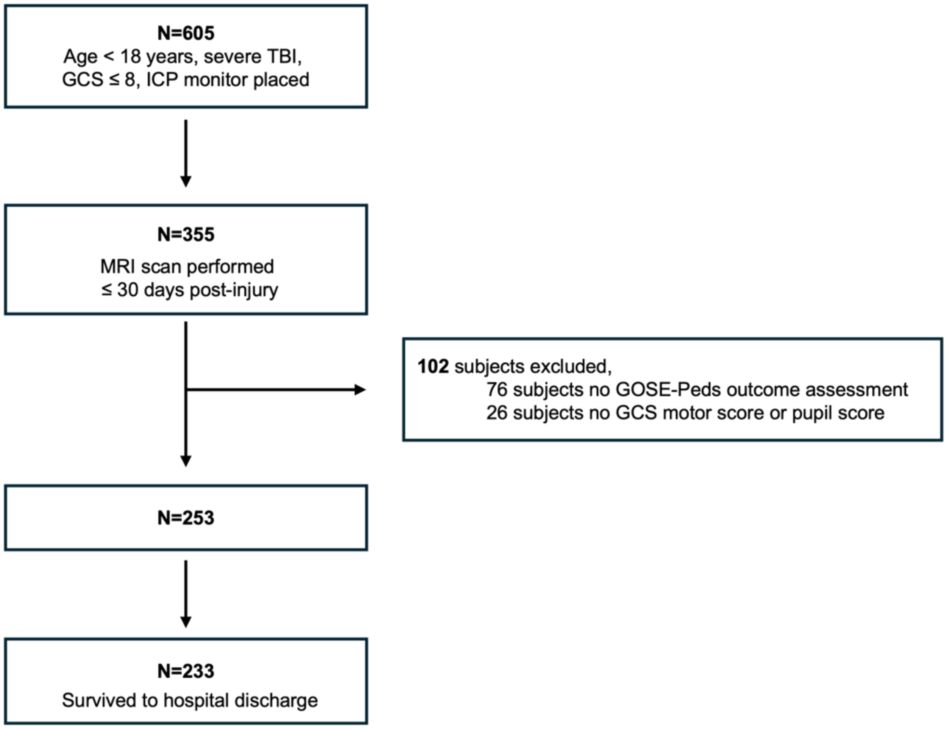

© 2024 Ferrazzano PA et al. *JAMA Network Open.*
