# Supplementary material for: MRI and Clinical Variables for Prediction of Outcomes After Pediatric Severe Traumatic Brain Injury
Source: JAMA Netw Open. 2024 Aug 5;7(8):e2425765. doi: 10.1001/jamanetworkopen.2024.25765 (PMC11301548; doi:10.1001/jamanetworkopen.2024.25765)
Supplement: Supplement 2. — Nonauthor Collaborators [file jamanetwopen-e2425765-s002.pdf]

Supplement 2. Nonauthor Collaborators
*First name, last name, and suffix (if applicable) are required and will appear in PubMed.

***Group Name(s): ADAPT MRI Investigators**							
***First Name and Middle Initial(s)**	***Last Name**	***Suffix (eg, Jr, III)**	**Academic Degrees**	**Institution**	**Location (city, state/province, country)**	**Role or Contribution, eg, chair, principal investigator**	**Group (if more than 1 Group listed in the byline) and/or Subgroup (eg, Steering Committee)**
Shruti	Agrawal		MD	Addenbrookes Hospital	Cambridge, UK	Site-PI	ADAPT MRI
Rachel	Agbeko		MD	The Newcastle Upon Tyne Hospitals NHS Foundation Trust	Newcastle, UK	Site-PI	ADAPT MRI
Warwick	Butt		MD	Murdoch Children's Research Institute, Royal Children's Hospital	Melbourne, Australia	Site-PI	ADAPT MRI
Ranjit S.	Chima		MD	Cincinnati Children's Hospital Medical Center	Cincinnati, OH	Site-PI	ADAPT MRI
Robert	Clark		MD	University of Pittsburgh	Pittsburgh, PA	Site-PI	ADAPT MRI
Mary	Hilfiker		MD	University of California	San Diego, CA	Site-PI	ADAPT MRI
Kerri	LaRovere		MD	Boston Children's Hospital	Boston, MA	Site-PI	ADAPT MRI
Iain	Macintosh		MD	University Hospital Southampton NHS Foundation Trust	Southampton, UK	Site-PI	ADAPT MRI
Darryl	Miles		MD	University of Texas Southwestern M	Dallas, TX	Site-PI	ADAPT MRI
Kevin	Morris		MD	Birmingham Children's Hospital NHS	Birmingham, UK	Site-PI	ADAPT MRI
Nicole	O'Brien		MD	Nationwide Children's Hospital	Columbus, OH	Site-PI	ADAPT MRI
Jose	Pineda		MD	Washington University–St. Louis	St. Louis, MO	Site-PI	ADAPT MRI
Courtney	Robertson		MD	Johns Hopkins University	Baltimore, MD	Site-PI	ADAPT MRI
Heather	Siefkes		MD	University of California, Davis	Sacramento, CA	Site-PI	ADAPT MRI
Neal	Thomas		MD	Pennsylvania State University	Hershey, PA	Site-PI	ADAPT MRI
Karen	Walson		MD	Children's Healthcare of Atlanta	Atlanta, GA	Site-PI	ADAPT MRI
Nico	West		MD	University of Tennessee	Memphis, TN	Site-PI	ADAPT MRI
Margaret	Winkler		MD	University of Alabama at Birminghan	Birmingham, AL	Site-PI	ADAPT MRI
Brandon	Zielinski		MD	University of Utah	Salt Lake City, UT	Site-PI	ADAPT MRI
Jerry	Zimmerman		MD	University of Washington	Seattle, WA	Site-PI	ADAPT MRI
