# Supplementary material for: MRI and Clinical Variables for Prediction of Outcomes After Pediatric Severe Traumatic Brain Injury
Source: JAMA Netw Open. 2024 Aug 5;7(8):e2425765. doi: 10.1001/jamanetworkopen.2024.25765 (PMC11301548; doi:10.1001/jamanetworkopen.2024.25765)
Supplement: Supplement 3. — Data Sharing Statement [file jamanetwopen-e2425765-s003.pdf]

Data Sharing Statement

Ferrazzano. MRI and Clinical Variables for Prediction of Outcomes After Pediatric Severe Traumatic Brain Injury. *JAMA Netw Open*. Published August 05, 2024. doi:10.1001/jamanetworkopen.2024.25765

Data
Data available: Yes
Data types: Deidentified participant data
How to access data: Study data is available in the Federal Interagency Traumatic Brain Injury Research (FITBIR) Informatics System: https://fitbir.nih.gov.
When available: With publication

Supporting Documents
Document types: None

Additional Information
Who can access the data: Anyone requesting and accessing data via FITBIR.
Types of analyses: Any purpose
Mechanisms of data availability: Without investigator support.
